# Maximal Holevo Quantity Based on Weak Measurements

**DOI:** 10.1038/srep10727

**Published:** 2015-06-19

**Authors:** Yao-Kun Wang, Shao-Ming Fei, Zhi-Xi Wang, Jun-Peng Cao, Heng Fan

**Affiliations:** 1 Beijing National Laboratory for Condensed Matter Physics, Institute of Physics, Chinese Academy of Sciences, Beijing 100190, China; 2 College of Mathematics, Tonghua Normal University, Tonghua, Jilin 134001, China; 3 School of Mathematical Sciences, Capital Normal University, Beijing 100048, China; 4 Max-Planck Institute for Mathematics in the Sciences, 04103 Leipzig, Germany; 5 Collaborative Innovation Center of Quantum Matter, Beijing 100190, China

## Abstract

The Holevo bound is a keystone in many applications of quantum information theory. We propose “ maximal Holevo quantity for weak measurements” as the generalization of the maximal Holevo quantity which is defined by the optimal projective measurements. The scenarios that weak measurements is necessary are that only the weak measurements can be performed because for example the system is macroscopic or that one intentionally tries to do so such that the disturbance on the measured system can be controlled for example in quantum key distribution protocols. We evaluate systematically the maximal Holevo quantity for weak measurements for Bell-diagonal states and find a series of results. Furthermore, we find that weak measurements can be realized by noise and project measurements.

Weak measurements was introduced by Aharonov, Albert, and Vaidman (AAV)^1^ in 1988. The standard measurements can be realized as a sequence of weak measurements which result in small changes to the quantum state for all outcomes^2^. Weak measurements realized by some experiments are also very useful for high-precision measurements^3^,^4^,^5^,^6^,^7^.

The quantum correlations of quantum states include entanglement and other kinds of nonclassical correlations. It is well known that the quantum correlations are more general than the well-studied entanglement^8^,^9^. Quantum discord, a quantum correlation measure differing from entanglement, is introduced by Oliver and Zurek^10^ and independently by Henderson and Vedral^11^. It quantifies the difference between the mutual information and maximum classical mutual information, i.e., it is a measure of the difference between total correlation and the classical correlation. Significant developments have been achieved in studying properties and applications of quantum discord. In particular, there are some analytical expressions for quantum discord for two-qubit states, such as for the 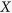 states^12^,^13^,^14^,^15^,^16^,^17^. Besides, researches on the dynamics of quantum discord in various noisy environments have revealed many attractive features^18^,^19^,^20^. It is demonstrated that discord is more robust than entanglement for both Markovian and non-Markovian dissipative processes. As with projection measurements, weak measurements are also applied to study the quantification of quantum correlation. For example, the super quantum correlation based on weak measurements has attracted much attention^21^,^22^,^23^,^24^,^25^.

In general, maximum classical mutual information is called classical correlation which represents the difference in von Neumann entropy before and after the measurements^11^. A similarly defined quantity is the Holevo bound which measures the capacity of quantum states for classical communication^26^,^27^. The Holevo bound is an exceedingly useful upper bound on the accessible information that plays an important role in many applications of quantum information theory^28^. It is a keystone in the proof of many results in quantum information theory^29^,^30^,^31^,^32^,^33^,^34^.

The maximal Holevo quantity for projective measurements (MHQPM) has been investigated^33^. Due to the fundamental role of weak measurements, it is interesting to know how MHQPM will be if weak measurements are taken into account. Recently, it is shown that weak measurements performed on one of the subsystems can lead to “super quantum discord” which is always larger than the normal quantum discord captured by projective measurements^21^. It is natural to ask whether weak measurements can also capture more classical correlations. In this article, we shall give the definition of “super classical correlation” by weak measurements as the generalization of classical correlation defined for standard projective measurements. As the generalization of MHQPM, we propose “ maximal Holevo quantity for weak measurements (MHQWM)”. Interestingly, by tuning continuously from strong measurements to weak measurements, the discrepancy between MHQWM and MHQPM becomes larger. Such phenomenon also exits between super classical correlation and classical correlation. In comparison with super quantum discord which is larger than the standard discord, MHQWM and super classical correlation becomes less when weak measurements are applied, while they are completely the same for projective measurements. In this sense, weak measurements do not capture more classical correlations. It depends on the specified measure of correlations. We calculate MHQPM for Bell-diagonal states, and compare the results with classical correlation. We give super classical correlation and MHQWM for Bell-diagonal states and compare the relations among super quantum correlations, quantum correlations, classical correlation, super classical correlation, and entanglement. The dynamic behavior of MHQWM under decoherence is also investigated.

## Results

### Maximal holevo quantity for projective measurements and weak measurements

The quantum discord for a bipartite quantum state *ρ*_*AB*_ with the projection measurements 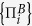 performed on the subsystem 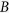 is the difference between the mutual information *I*(*ρ*_*AB*_)^35^ and classical correlation 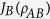
11:


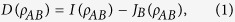


where


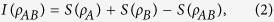



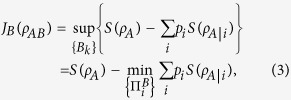


with the minimization going over all projection measurements 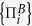, where 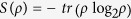 is the von Neumann entropy of a quantum state *ρ*, *ρ*_*A*_, *ρ*_*B*_ are the reduced density matrices of *ρ*_*AB*_ and


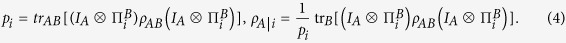


The Holevo quantity of the ensemble 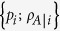
33 that is prepared for A by B via B’s local measurements is given by


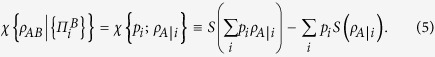


It denotes the upper bound of A’s accessible information about B’s measurement result when B projects its system by the projection operaters 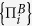. The Maximal Holevo quantity for projective measurements (MHQPM)^33^ of the state *ρ*_*AB*_ over all local projective measurements on B’s system, denoted by *C*_1_(*ρ*_*AB*_), is defined as


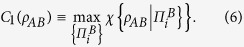


The weak measurement operators are given by^2^


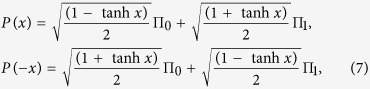


where *x* is the measurement strength parameter, 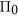 and 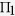 are two orthogonal projectors with 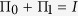. The weak measurement operators satisfy: (i) 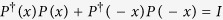, (ii) 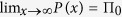 and 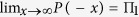.

Recently, super quantum discord for bipartite quantum state *ρ*_*AB*_ with weak measurements on the subsystem 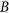 has been proposed^21^. Similarly to the definition of quantum discord, we give another form of definition of super quantum discord. We define super classical correlation 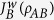 for bipartite quantum state *Ρ*_*AB*_ with the weak measurements 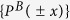 performed on the subsystem *B* as follow. The super quantum discord denoted by *D*_*w*_(*ρ*_*AB*_) is the difference between the mutual information *I*(*Ρ*_*AB*_) and super classical correlation 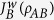, i.e.,


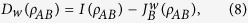


where


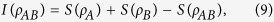



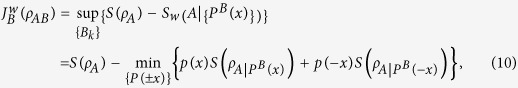


with the minimization going over all weak measurements,


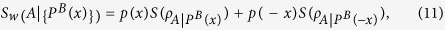



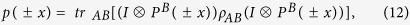



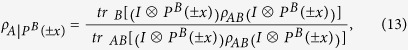


where 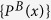 is weak measurement operators performed on the subsystem *B*.

Now, let us define the Holevo quantity of the ensemble 
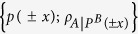
 for weak measurements on the subsystem *B*,


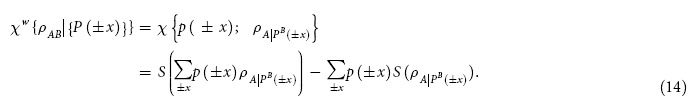


It denotes the upper bound of A’s accessible information about B’s measurement results when B projects the system with the weak measurements operaters 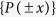. We define maximum value of the Holevo quantity over all local weak measurements on B’s system to be the maximal Holevo quantity for weak measurements (MHQWM). MHQWM denoted by 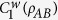, is given by


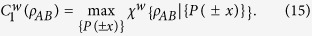


Next, we consider MHQPM and MHQWM for two-qubit Bell-diagonal states,


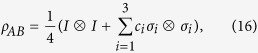


where *I* is the identity matrix, −1 ≤ c_*i*_ ≤ 1. The marginal states of *ρ*_*AB*_ are 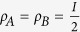. The MHQPM for Bell-diagonal states is given as


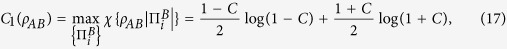


where 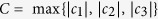. We find that MHQPM *C*_1_(*ρ*_*AB*_) equals to the classical correlation *J*_*B*_(*ρ*_*AB*_),


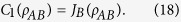


The MHQWM of two-qubit Bell-diagonal states is given by


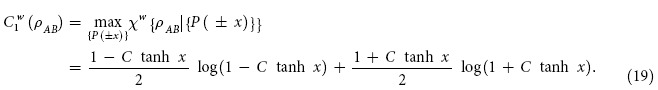


The super classical correlation of two-qubit Bell-diagonal states is given by


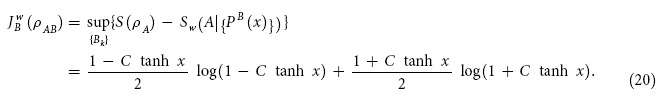


MHQWM 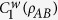 equals to super classical correlation 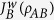, i.e.,


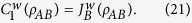


Then, we compare MHQWM (super classical correlation), MHQPM (classical correlation), super quantum discord, quantum discord, and entanglement of formation. For simplicity, we choose Werner states, *c*_1_ = *c*_2_ = *c*_3_ = −*z*,


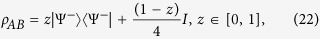


where 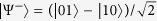. Set 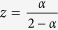. The Werner states have the form


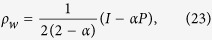


where −1 ≤ *α *≤ 1, *I* is the identity operator in the 4-dimensional Hilbert space, and 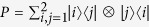 is the operator that exchanges A and B. The entanglement of formation *E*_*f*_ for the Werner states is given as 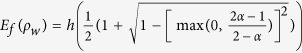, by 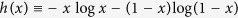. The MHQPM for werner states is given by, see Eq. (48) in section Method,


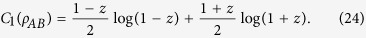


The MHQWM for werner states is given by, see Eq. (57) in section Method,


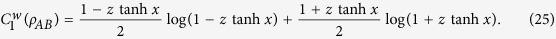


Quantum discord for Werner states is given by^12^


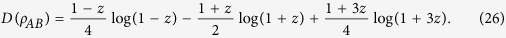


Super quantum discord for Werner states is given by^21^


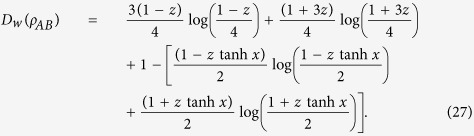


**Figure 1:**
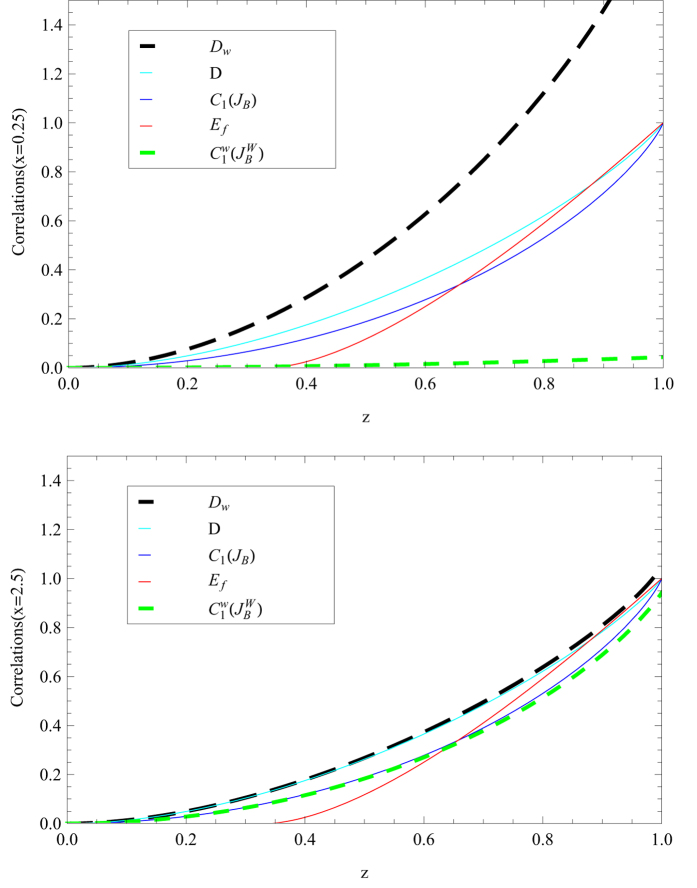
MHQWM (super classical correlation) (dashed green line), MHQPM (classical correlation) (solid blue line), quantum discord(solid cyan line), super quantum discord (dashed black line), and entanglement of formation(solid red line) for the Werner states as a function of *z*: *x* = 0.25 and *x* = 2.5.

In Fig. 1 we plot MHQWM, MHQPM, super quantum discord, quantum discord, and entanglement of formation for Werner states. We find that super quantum discord , quantum discord, MHQPM and MHQWM have the relation, 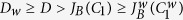. For the case of projection measurements, 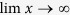, we have 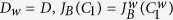. MHQWM approaches to zero for smaller values of 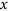. MHQWM approaches to MHQPM and super quantum discord approaches to quantum discord for larger values of *x*. MHQWM and MHQPM are larger than the entanglement of formation for small *z* and smaller than the entanglement of formation for big *z*. It shows that MHQWM and MHQPM can not always capture more correlation than the entanglement as super quantum discord and quantum discord do.

As a natural generalization of the classical mutual information, the classical correlation represents the difference in von Neumann entropy before and after projection measurements, i.e.,


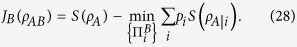


Similarly, the super classical correlation represents the difference in von Neumann entropy before and after weak measurements, i.e.,


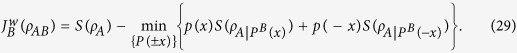


As weak measurements disturb the subsystem of a composite system weakly, the information is less lost and destroyed by weak measurements on the subsystem alone. That is the physical interpretation that the super classical correlation is smaller than the classical correlation, 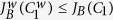. According to this fact, we can infer that weak measurements can capture more quantum correlation than projection measurements. In fact, the super quantum correlation 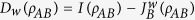 is lager than the quantum correlation 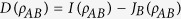. There is a similarity to the Holevo quantity which measures the capacity of quantum states for classical communication.

### Dynamics of MHQWM of Bell-diagonal states under local nondissipative channels.

We will consider the system-environment interaction^28^ through the evolution of a quantum state *ρ* under a trace-preserving quantum operation *ε*(*ρ*),


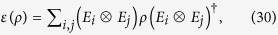


where 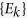 is the set of Kraus operators associated to a decohering process of a single qubit, with 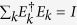. We will use the Kraus operators in Table 1^36^ to describe a variety of channels considered in this work.

The decoherence processes BF, PF, and BPF in Table 1 preserve the Bell-diagonal form of the density operator *ρ*_*AB*_. For the case of GAD, the Bell-diagonal form is kept for arbitrary *γ* and *p* = 1/2. In this situation, we can write the quantum operation ε(*ρ*) as


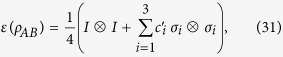


where the values of the 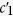, 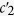, 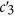 are given in Table 2^36^.

When 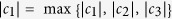, 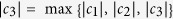, 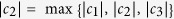, respectively, we have that 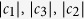 are the maximal values among 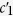, 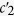, 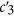 in each line of Tabel 2 . As ε(*ρ*) are also Bell-diagonal states, from Eqs. (46), (48), (49), (57), (58) we find that classical correlation, MHQPM, super classical correlation, and MHQWM for Bell-diagonal states through any channel of bit flip, phase flip, bit-phase flip remain unchanged. In particular, for Werner states, we find that classical correlation, MHQPM, super classical correlation, and MHQWM for Werner states keep unchanged under all channels of bit flip, phase flip, bit-phase flip.

The MHQPM of the Werner states under generalized amplitude damping is given by


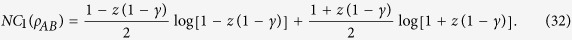


The MHQWM of the Werner states under generalized amplitude damping is given by


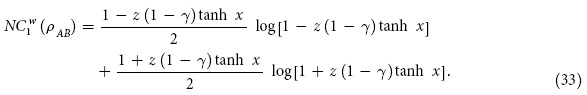


**Figure 2:**
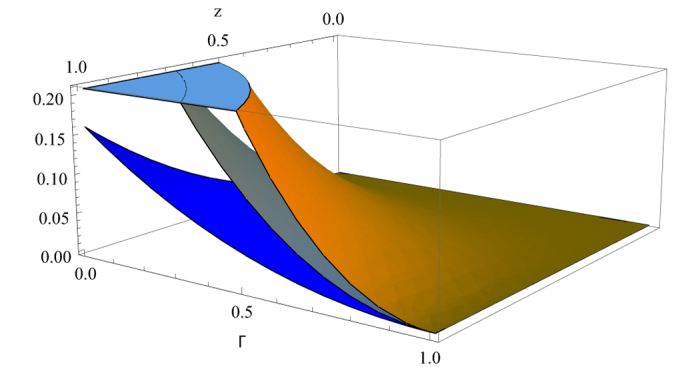
The MHQWM (super classical correlation) {x = 0.5 (blue surface), *x* = 1(gray surface)} and the MHQPM (classical correlation)(orange surface) for the Werner states under generalized amplitude damping channel as a function of *z* and γ.

In Fig. 2, as an example, the dynamic behaviors of the MHQWM and MHQPM for the Werner states under the generalized amplitude damping channel are depicted for *x* = 0.5 and *x* = 1. Against the decoherence, when *x* increases, MHQWM become greater. MHQWM approaches to MHQPM for larger *x* under the generalized amplitude damping channel. MHQWM and MHQPM increase as *z* increases. Then as *γ* increases, MHQWM and MHQPM decrease.

### Weak measurements can be realized by noise and project measurements

Now we study the realization of weak measurements by means of depolarizing noise and project measurements. The depolarizing noise is an important type of quantum noise that transforms a single qubit state into a completely mixed state *I*/2 with probability *p* and leaves a qubit state untouched with probability 1 − *p*. The operators for single qubit depolarizing noise are given by^37^


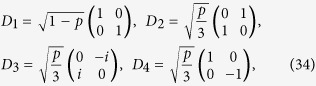


where *p* = 1−*e*^−*τt*^. Then the Bell-diagonal states under the depolarizing noise acting on the first qubit of quantum state *ρ*_*AB*_ are given by^37^


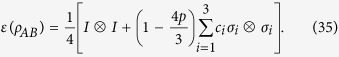


As *ε*(*ρ*_*AB*_) is also a Bell-diagonal state, after projective measurements on *B*, see Eq. (41) in section Method, the state *ε*(*ρ*_*AB*_) becomes the following ensemble with 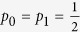 and


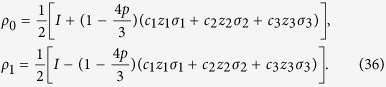


Comparing Eq. (36) with the ensemble after weak measurements Eq. (52) in section Method, when 
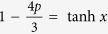
, we obtain that weak measurements can be realized by means of depolarizing noise and projective measurements.

## Discussion

We have evaluated analytically MHQPM for Bell-diagonal states and find that it equals to the classical correlation. We have given the definition of “super classical correlation” by weak measurements as the generalization of classical correlation defined by standard projective measurements. We have evaluated super classical correlation for Bell-diagonal states and find that it is smaller than the classical correlation and approaches the classical correlation by tuning the weak measurements continuously to the projective measurements. We have shown the physical implications that weak measurements can capture more quantum correlation than projective measurements.

As the generalization of the MHQPM defined by projective measurements, we have also proposed MHQWM by weak measurements. We have evaluated MHQWM for Bell-diagonal states and find that it is smaller than MHQPM in general. Moreover, it has been shown that MHQWM equals to super classical correlation.

As applications, the dynamic behavior of the MHQWM under decoherence has been investigated. For some special Bell-diagonal states, we found that MHQWM remain unchanged under all channels of bit flip, phase flip and bit-phase flip.

The dynamical behaviors of the MHQWM for Werner states under the generalized amplitude damping channel have been investigated. Under the generalized amplitude damping channel, MHQWM becomes greater when *x* increases and approaches to MHQPM for larger *x*. MHQWM increases as *z* increases. MHQWM decreases as *γ* increases. Above all, it has been shown that weak measurements can be realized by means of depolarizing noise and projective measurements.

The Holevo bound is a keystone in quantum information theory and plays important roles in many quantum information processing. While MHQPM provides us different perspectives about classical correlations. The behaviors of the MHQWM vary a lot with the strength of the weak measurements. Those measures can be applied to various protocols in quantum information processing, and identify the importance of the classical correlations in those protocols.

## Methods

### Calculation of the MHQPM for Bell-diagonal states.

We compute the MHQPM *C*_1_(*ρ*_AB_) of Bell-diagonal states. Let 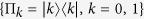 be the local measurements on the system *B* along the computational base 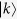. Any von Neumann measurement on the system *B* can be written as


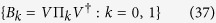


for some unitary *V* ∈ *U*(2). Any unitary *V* can be written as


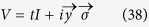


with 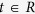, 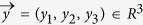, and 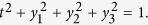 After the measurements *B*_*k*_, the state *ρ*_*AB*_ will be changed to the ensemble 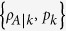 with


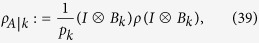



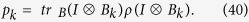


After some algebraic calculations^12^, we obtain 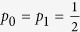 and


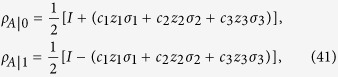


where


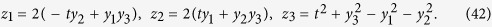


Therefore,


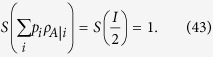


Denote 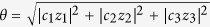. Then


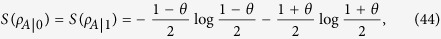


and


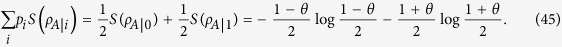


It can be directly verified that 
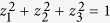
. Let


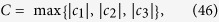


then we have 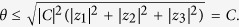 Hence we get 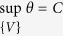 and *θ* ∈ [0, *C*]. It can be verified that 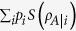 is a monotonically decreasing function of *θ* in the interval of 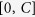. The minimal value of 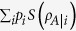 can be attained at the point *C*,


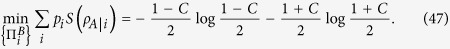


By Eqs. (43) and (47), we obtain


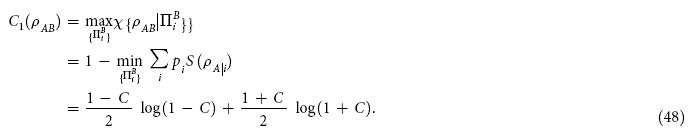


As 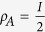, the classical correlation *J*_*B*_(*ρ*_*AB*_) is given by


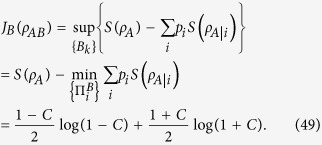


### Calculation of the MHQWM for Bell-diagonal states

Let 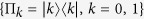 be the local measurements for the part *B* along the computational base 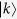. Then any weak measurement operators on the system *B* can be written as


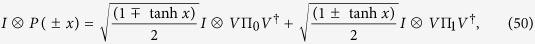


for some unitary *V* ∈ U(2) of the form Eq. (38).

After weak measurements the resulting ensemble is given by 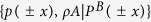. We need to evaluate 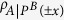 and *p*(+*x*). By using the relations^12^,


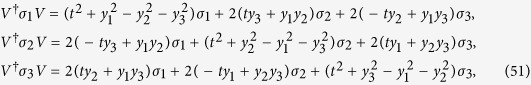


and 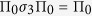, 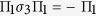, 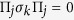 for 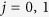, 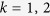, from Eqs. (12) and (13), we obtain 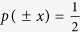 and


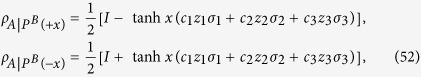


where 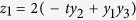, 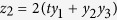 and 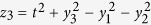. Therefore, we see that


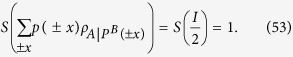


Denote 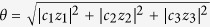. Then


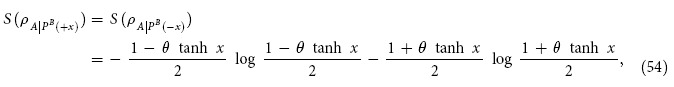


and


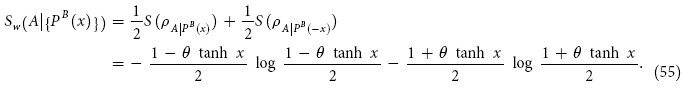


Let 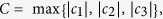 then 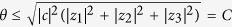. Hence we get 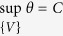 and *θ* ∈ [0, *C*]. It can be verified that 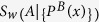 is a monotonically decreasing function of *θ* in the interval of [0, *C*]. The minimal value of 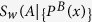 can be attained at point *C*,


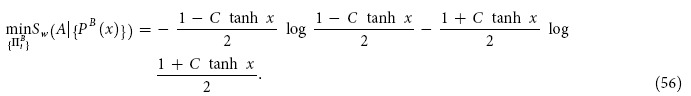


By Eqs. (53) and (56), we obtain


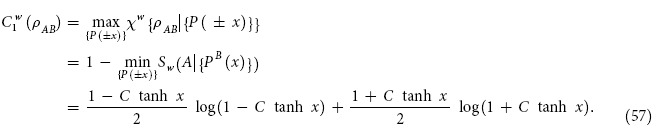


As 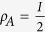, the super classical correlation 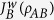 is given by


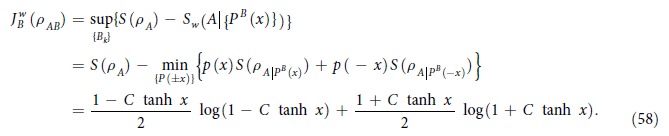


## Additional Information

**How to cite this article**: Wang, Y.-K. *et al*. Maximal Holevo Quantity Based on Weak Measurements. *Sci. Rep*. **5**, 10727; doi: 10.1038/srep10727 (2015).

## Figures and Tables

**Table 1:** Kraus operators for the quantum channels: bit flip (BF), phase flip (PF), bit-phase flip (BPF), and generalized amplitude damping (GAD), where *p* and *γ* are decoherence probabilities, 0 < *p* < 1, 0 < *γ* < 1.

	Kraus operators
BF	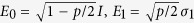
PF	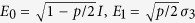
BPF	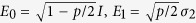
GAD	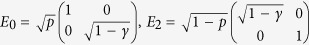
	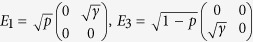

**Table 2:** Correlation functions for the quantum operations: bit flip (BF), phase flip (PF), bit-phase flip (BPF), and generalized amplitude damping (GAD). For GAD, we fixed *p* = 1/2.

Channel	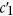	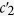	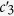
BF	*c*_1_	*c*_2_(1−*p*)^2^	*c*_3_(1−*p*)^2^
PF	*c*_1_(1−*p*)^2^	*c*_2_(1−*p*)^2^	*c*_3_
BPF	*c*_1_(1−*p*)^2^	*c*_2_	*c*_3_(1−*p*)^2^
GAD	*c*_1_(1−*γ*)	*c*_2_(1−*γ*)	*c*_3_(1−*γ*)^2^
